# Partially Acetylated Cellulose Dissolved in Aqueous Solution: Physical Properties and Enzymatic Hydrolysis

**DOI:** 10.3390/polym11111734

**Published:** 2019-10-23

**Authors:** Gilad Alfassi, Dmitry M. Rein, Avi Shpigelman, Yachin Cohen

**Affiliations:** 1 Department of Biotechnology Engineering, ORT Braude College, Karmiel 2161002, Israel; 2 Faculty of Chemical Engineering, Technion-Israel Institute of Technology, Haifa 3200003, Israel; cerycdr@technion.ac.il (D.M.R.); yachinc@technion.ac.il (Y.C.); 3 Faculty of Biotechnology & Food Engineering, Technion-Israel Institute of Technology, Haifa 3200003, Israel; avis@bfe.technion.ac.il

**Keywords:** water soluble, cellulose acetate, polymer solution properties, enzymatic hydrolysis

## Abstract

Cellulose acetate is one of the most important cellulose derivatives. The use of ionic liquids in cellulose processing was recently found to act both as a solvent and also as a reagent. A recent study showed that cellulose dissolution in the ionic liquid 1-ethyl-3-methylimidazoliumacetate (EMIMAc) mixed with dichloromethane (DCM) resulted in controlled homogenous cellulose acetylation; yielding water-soluble cellulose acetate (WSCA). This research investigated the properties of cellulose acetate prepared in this manner, in an aqueous solution. The results revealed that WSCA fully dissolves in water, with no significant sign of molecular aggregation. Its conformation in aqueous solution exhibited a very large persistence length, estimated as over 10 nm. The WSCA exhibited surface activity, significantly reducing the surface tension of water. Because of the molecular dissolution of WSCA in water, augmented by its amphiphilicity, aqueous solutions of WSCA exhibited an overwhelmingly high rate of enzymatic hydrolysis.

## 1. Introduction

Cellulose acetate (CA), formed by partial or full acetylation of the three hydroxyl groups of anhydroglucose (AGU), the repeat unit of cellulose, is one of the oldest synthetic materials, studied since the end of the nineteenth century. Cellulose triacetate (degree of substitution DS~3) is usually manufactured by a direct irreversible cellulose acetylation reaction using acetic anhydride. The triacetate product can be subjected to partial hydrolysis with dilute sulfuric acid, to yield secondary acetates with a lower degree of substitution (DS), e.g., monoacetate (DS~1) and diacetate (DS~2). It is well-known that cellulose acetates with DS in the range of 0.4–0.9 are water-soluble [1,2,3,4]. Unfortunately, undesired degradation of the cellulose chain occurs during the deacetylation process [2]. A number of additional drawbacks are associated with this procedure rendering it commercially unattractive. These include long reaction times (8–48 h) at relatively high temperatures (140–160 °C) and the need for continuous or sequential addition of water to maintain reaction rates yielding dilute reaction mixtures (often <5% w/w), which require significant use of energy for subsequent solvent recycling [4,5].

The physical properties of CAs are determined by the conformational behavior of the chains and their intermolecular interactions. Chemically homogeneous sequences of un-derivatized or fully derivatized glucopyranose units can locally organize into a crystalline structure; whereas the heterogeneous ones comprised of differently substituted monomers randomly distributed along the chain, are amorphous [6,7]. X-ray diffraction observations revealed that the water-soluble CA is essentially noncrystalline [3]. CA samples merely presented a broad peak around the scattering angle 2*θ* = 20.0 degree, confirming that the original crystalline structure of cellulose was largely destroyed [8,9]. At the same time, it has been reported that the water solubility of CA depends on both the overall DS, as well as on the distribution of acetyl substituents along the chain. Substitution of secondary hydroxyls at C-2 and C-3 of AGU by acetyl groups plays a key role in the water solubility of CA [3,10]. Although the acetyl groups are less hydrophilic than the hydroxyl groups, their presence decreases the intra and inter molecular H-bonds along the cellulose chain, thus rendering it water soluble within a certain range of acetyl group substitution (DS 0.4–0.9). However, a further increase in acetyl substitution (DS > 0.9) increases the hydrophobicity of the cellulose chain, rendering it insoluble in water [11].

Innovations in cellulose solvents motivated the quest for new acetylation procedures that can enable homogenous acetylation in ionic liquids (IL), in particular 1-ethyl-3-methylimidazolium acetate (EMIMAc) [12,13,14], which promote unique cellulose solution behavior. Not only was EMIMAc found to dissolve cellulose to individual chains, it also proved less toxic and is biodegradable [13,15], which renders it an excellent solvent for downstream processing [16,17,18] and cellulose derivatization. On the other hand, it was reported that cellulose dissolution in 1-ethyl-3-methylimidazolium chloride-(EMIMCl) resulted in significant cellulose depolymerization [19]. In some cases, dissolving cellulose in IL with a co-solvent results in acetylation, such as in the case of a mixture of EMIMAc with bulky chlorides (*p*-toluenesulfonyl chloride, triphenylmethyl chloride, or 2-furoyl chloride) [20]. Water-soluble cellulose acetate (WSCA) was synthesized by adding dichloroacetyl chloride to a cellulose solution in a carboxylate ionic liquid such as EMIMAc [8]. Our previous work demonstrated the ability of dichloromethane (DCM), a simple and relatively small chloro-organic co-solvent, mixed with the ionic liquid EMIMAc, to facilitate direct homogenous acetylation. The DCM content controlled the degree of substitution, such that direct formation of WSCA was achieved. The work described details the reaction mechanism, which involves a methylene diacetate or chloromethyl acetate intermediate, formed by nucleophilic substitution of DCM, as the acetylation reagent [9].

ILs can serve as efficient solvents in biomass valorization, in particular processes that include enzymatic cellulose hydrolysis [21,22,23]. Pretreatment by IL disrupts the strong inter-chain interactions in the native cellulose crystal structure, and subsequent regeneration by a non-solvent such as water yields a larger and more accessible surface area for hydrolyzing enzymes [24,25,26,27]. It is thus of interest to evaluate the ultimate hydrolysis of cellulose fully dissolved in aqueous solution.

The objectives of this work were to evaluate some of the physical properties of WSCA prepared using EMIMAc and DCM, in particular the chain conformation and extent of aggregation in water, and to assess the amphiphilicity of the dissolved WSCA by its reduction of water surface tension. Moreover, we pose the hypothesis that molecular dissolution coupled with amphiphilicity lead to an overwhelmingly high rate of enzymatic cellulose hydrolysis.

## 2. Materials and Methods

### 2.1. Materials

Microcrystalline cellulose (MCC) powder (Avicel^®^), with particle size in the range of 70–250 µm, was purchased from Sigma-Aldrich Co., (Rehovot, Israel). EMIMAc of 96% purity, was supplied by Iolitec (Heilbronn, Germany). DCM was purchased from Bio-Lab Ltd (Jerusalem, Israel). EMIMAc and cellulose were dried in a vacuum oven (MRC, Holon, Israel) at 60 °C, 0.26 KPa, for at least 24 h.

### 2.2. Sample Preparation

Dissolution and acetylation of cellulose were performed in two stages. First, 0.8 g of cellulose powder was soaked in 0.6 g ice-cooled DCM and then 19.2 g EMIMAc was immediately added to the mixture, and was placed in a shaker incubator (MRC, Holon, Israel), at 70 °C and 150 rpm, for at least 12 h, until no cellulose crystals were visually observed [9]. This was carried out in 20-mL closed vials to avoid DCM evaporation. The remaining DCM, if any, was subsequently evaporated in a vacuum oven at 60 °C, 0.26 KPa, for at least 24 h [9]. The resultant solution was dialyzed against deionized water for 24 h, using dialysis tubing (Spectrum Laboratories, Inc., Spectra/Por 4 Regenerated Cellulose, MWCO 12–14 kDa, Rancho Dominguez, CA, USA filled by 20 mL solution), until electrical conductivity of the dialyzing fluid was below 1 mS/cm. The concentration of the WSCA extracted from the dialysis was determined gravimetrically. Different concentrations of WSCA were prepared by adding deionized water to the WSCA extracted for the dialysis.

Enzymatic hydrolysis was performed using Accellerase 1500 (DuPont, Rochester, NY, USA), at 30 FPU/g and a shaker incubator at 50 °C, 120 rpm as described earlier [28]. The amount of cellulose converted to fermentable sugars was determined using dinitrosalicylic acid (DNS, Sigma-Aldrich Co., Rehovot, Israe) in a redox reaction [29].

### 2.3. Equipment and Characterization Methods

Small-angle x-ray scattering (SAXS) measurements were performed using a molecular metrology SAXS/WAXS system (Rigaku Innovative Technologies, Auburn Hills, MI. USA) equipped with a sealed microfocus tube emitting CuK_α_ radiation (MicroMax-002+S), two Gӧbel mirrors, three pinhole slits, and a generator powered at 45 kV and 0.9 mA. The scattering patterns were recorded by a two-dimensional position sensitive wire detector (Gabriel) positioned 150 cm behind the sample. The solutions were sealed in thin-walled glass capillaries about 2 mm in diameter and 0.01 mm wall thickness, and measured under vacuum at 25 °C. The scattered intensity I(q) was recorded as a function of the scattering vector q defined as: q = (4π/λ) sin(θ), where 2θ is the scattering angle, and λ is the radiation wavelength, at the interval 0.07 < q < 2.6 nm^−1^. The scattering intensity was normalized with respect to time, solid-angle, primary beam intensity, capillary diameter, and transmission, to yield the absolute specific scattering cross-section. The rheological properties of the solutions under steady-state shear flow were characterized using a Discovery DHR-2 rotational rheometer (TA Instruments, New Castle, DE. USA) equipped with cone-plate geometry (40 mm diameter, 0.58 cone angle) at 25 °C. Characterization of CA dissolved in water was performed using a size-exclusion chromatography instrument (SEC-MALLS-UV-RI) equipped with a multi-angle laser light scattering detector (MALLS, PN3609), refractive index detector (RI, PN3150) and UV detector, all from Postnova Analytics (Landsberg, Germany). Size exclusion separation was carried out at 35 °C, in a series of three waters columns (Waters, Milford, MA), namely, Ultra-hydrogel 250, 1000, and 2000, with exclusion limits of 8 × 10^4^, 4 × 10^6^, and 1 × 10^7^ g/mol, respectively. The water-soluble samples were prepared with 0.1 M NaNO_3_ and filtered (0.22 μm) before injection. The specific refractive index increment (dn/dc) that was used to calculate the concentration from the RI detector was determined as 0.141 mL/g, as measured by a differential refractometer using four concentrations, which is in the range of values for many polysaccharides in aqueous solutions [30]. The injection volume and flow rate were set on 100 μL and 0.5 mL/min respectively. The molecular weight was calculated using random coil fitting method (which was chosen as the appropriate model by Auto fitting method) by the MALLS detector software (Nova Malls, version 1.5.0.7, Postnova analytics, Landsberg, Germany). The surface tension of aqueous WSCA solutions was measured through a pendant drop method using a surface tension apparatus (OCA 15 PLUS, Data-Basics, Inc., Filderstadt, Germany) with integrated software (SCA 22, Filderstadt, Germany)). The instrument records the geometry of drops formed by solutions of various concentrations, and the software calculates the surface tension using the Young-Laplace equation. The reference liquid was deionized water and the surrounding of the drop was air.

## 3. Results and Discussion

**Figure 1:**
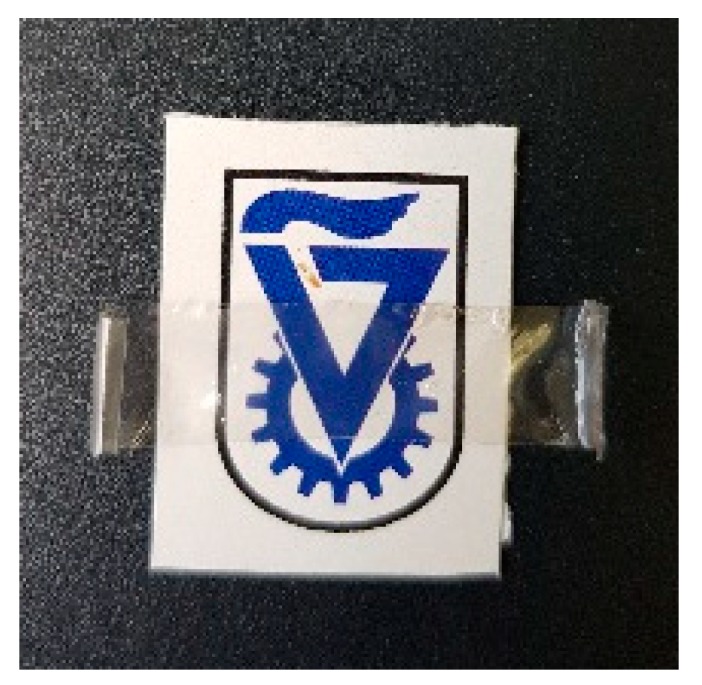
Water soluble cellulose acetate film.

The unique reaction of DCM with EMIMAc yields an intermediate that can derivatize cellulose to different DSs, as described in a previous publication [9]. Mixing 2—4 wt% DCM with EMIMAc, to serve as a solvent for cellulose, resulted in cellulose acetylation upon dissolution and enabled the formation of cellulose acetate with 15—23 % acetyl content, that is equivalent to DS of 0.4—0.9. At such low DS, cellulose is considered water-soluble. Replacement by dialysis of the DCM/EMIMAc solvent with water yields a clear aqueous solution. Evaporation of water from this solution resulted in the formation of a transparent soluble film, as shown in Figure 1. This film can be readily re-dissolved in water.

**Figure 2:**
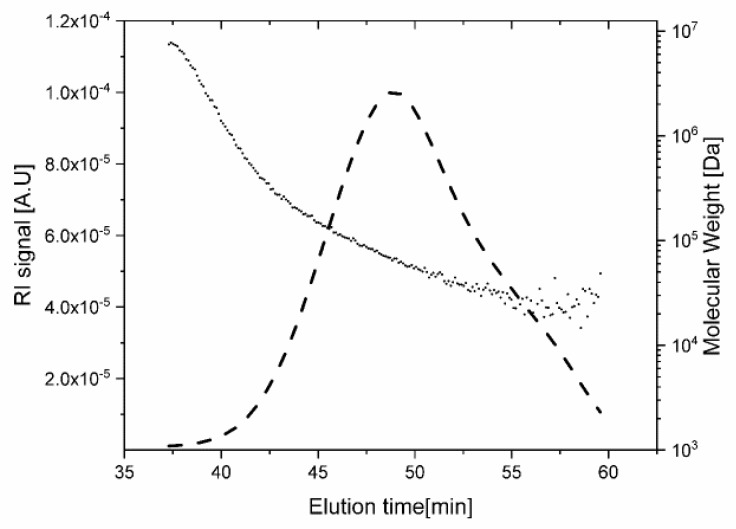
SEC elution profile of cellulose acetate dissolved in water (0.1 M NaNO_3_). Dashed line—RI signal (axis at left); dotted line molecular weight (axis at right).

Previous works that studied molecular weight distribution of CA usually did not use aqueous solutions [31,32,33,34]. The SEC chromatogram, shown in Figure 2, demonstrates that the cellulose molecules were not degraded by the dissolution and derivation processes. The molecular weights determined by chromatography (*M*_W_ average 50 kDa by number and 80 kDa by weight) were compatible with those of the cellulose precursor measured by light scattering from solutions in EMIMAC mixed with dimethylformamide [13], as well as those indicated by the manufacturer. The SEC chromatogram also shows that the derivatized cellulose is readily dissolved in water, with negligible sign of aggregates.

**Figure 3:**
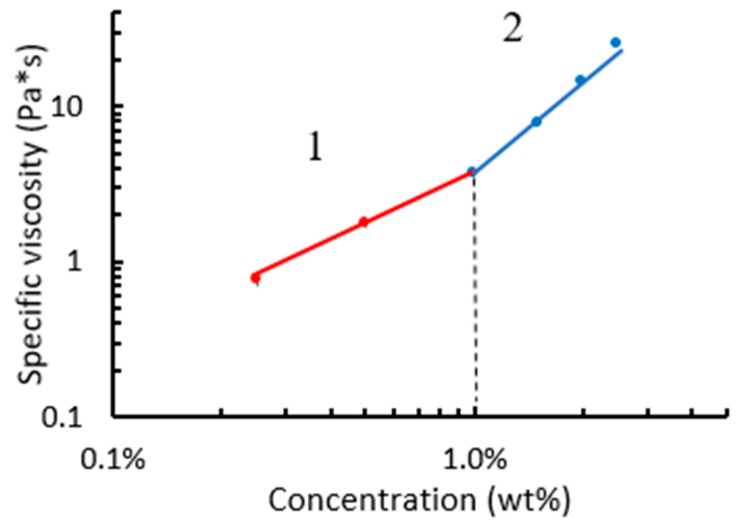
Dependence of the specific viscosity on cellulose acetate solutions concentration in water at 25 °C, with power-law fits. The dashed line represents the overlap concentrations c*.

The viscosity of aqueous WSCA solutions was measured in steady shear experiments. The specific viscosity, defined as η_sp_ = (η − η_s_)/η_s_ where η and η_s_ are the zero-shear viscosities of the solution and solvent (water), respectively, is presented in Figure 3 as a function of the solution concentration. Two different power-law relation regimes of specific viscosity to concentration can be noted. A linear regime (power exponent k~1) is observed at concentrations from 0.25 wt% to about 1.0 wt%, and a nonlinear regime is seen at concentrations of about 1.0–2.5 wt%, for which k~2.0. The former regime is typical of dilute solutions of neutral polymers, whereas the latter is characteristic of a semi-dilute un-entangled polymer solution at so-called “theta” solvent conditions. These measurements indicate that the water acts as a theta solvent for these cellulose acetate polymers, where the overlap concentration c* is about 1.0 wt%.

**Figure 4:**
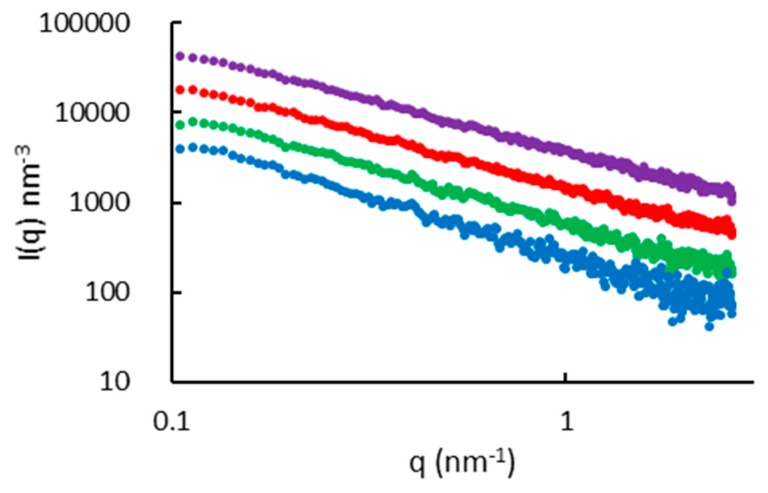
SAXS patterns of WSCA solutions in water, at concentrations, from bottom to top, of 1, 1.5, 2, 2.5 wt% (the plots have been spaced for clarity: 1.5% by 2, 2% by 3 and 2.5% by 5).

**Figure 5:**
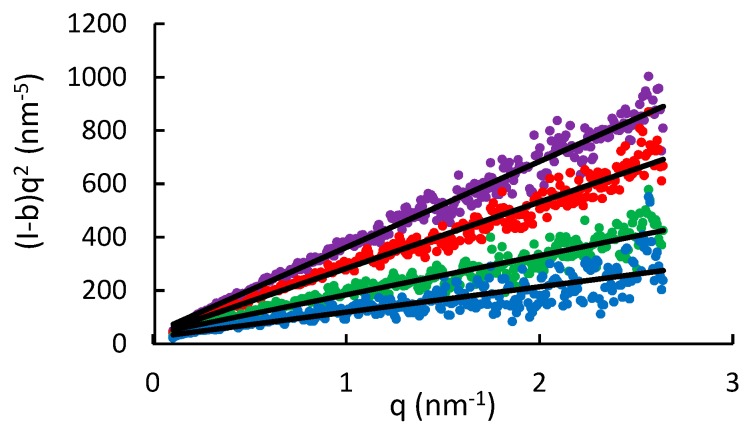
Linear fitting of SAXS patterns of aqueous WSCA solutions (Equation 2), at concentrations, from bottom to top, of 1, 1.5, 2, 2.5 wt%.

Figure 4 shows the SAXS patterns of aqueous WSCA solutions at four concentrations (1, 1.5, 2 and 2.5 wt%), at 25 °C. The information from the SAXS curves was extracted by fitting the data in the experimentally significant q range (0.01–0.25 Å^−1^). The patterns were fitted by the power law relation of Equation (1). Background subtraction allows a linear fit to Equation (2) as shown in Figure 5:

(1) $$I(q)=\frac{I_{0}^{}}{q}+b$$

(2) $$(I-b)q^{2}=I_{0}q$$

where *I*_0_ is the pre-factor of the power-law relation and *b* the background scattering. The q^−1^ intensity to scattering vector relation is characteristic of rod-like entities, and is typically observed in the range of scattering vectors (1/L) < *q* < (1/Rc), where *L* and *R*c represent the rod length and the gyration radius of its cross-section, respectively.

In this case, the pre-factor, *I*_0_ is given by [14]:

(3) $$I_{0}=\left(\frac{N}{V}\right)\pi LA^{2}\Delta \rho^{2}=\varphi \pi A\Delta \rho^{2}$$

where (*N*/*V*) is the number of rod-like particles per unit volume, *A* and φ are the rod cross-section area and volume fraction, respectively, and *Δρ* is the difference in scattering length density (SLD) between the rod and its surrounding medium. The values of *I*_0_ were obtained by fitting Equation (2) to the data in Figure 5, and the volume fractions of cellulose (*φ*), were calculated by Equation (3). For this calculation, the SLD of acetylated cellulose was calculated from the original cellulose parameters considering that at low DS, the values are similar to those of cellulose. Thus, SLD of 13.52 × 10^−4^ nm^−2^ was used, based on the cellulose formula (C_6_H_10_O_5_) with density of 1.5 g/cm^3^. The SLD of H_2_O is 9.47 × 10^−4^ nm^−2^, and a constant cross-section area *A* of 0.32 nm^2^ was used, as given for a chain in the cellulose I crystal structure [14]. The “experimental” values of the cellulose concentration (wt%), are compared in Table 1 with the “calculated” values determined from the volume fractions (*φ*) assuming a cellulose density of 1.5 g/cm^3^. 

The correspondence of the calculated and experimental φ values verified the validity of Equation (3) and confirmed the extended rod-like chain conformation of WSCA in water. A lower limit estimate of the persistence length (a) can be evaluated by the lowest q value of the linear fit (*q*_min_), as $a=1.91/q_{\min}$, yielding a value of 11 nm. It also validated the SEC results, by showing no aggregation (no excess scattering) and indicated full dissolution of the cellulose molecules in water. This result also showed that no significant degradation occurred during dissolution and acetylation.

**Figure 6:**
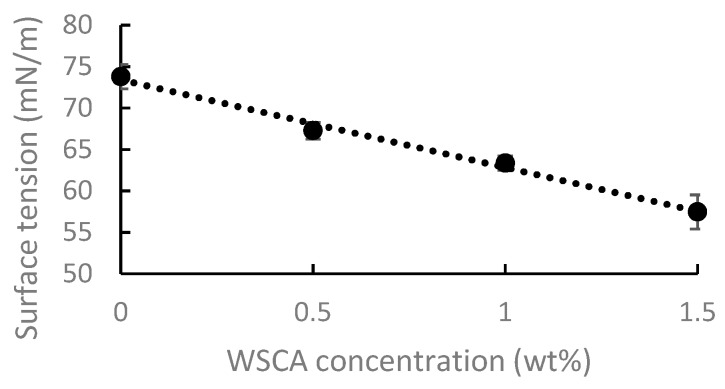
The effect of WSCA concentration on surface tension of water.

The structure of the cellulose chain is amphiphilic [35]. WSCA is expected to be even more amphiphilic because of the partial acetylation. It is thus of interest to evaluate its activity in reducing the surface tension of water. The effect of WSCA concentration on the surface tension of water was measured as shown in Figure 6. The results show a decrease in surface tension with increasing WSCA concentration. It is noted that the extent to which WSCA reduces the surface tension of water is similar to that of poly(vinyl pyrrolidone), a well-known water soluble amphiphilic polymer [36].

**Figure 7:**
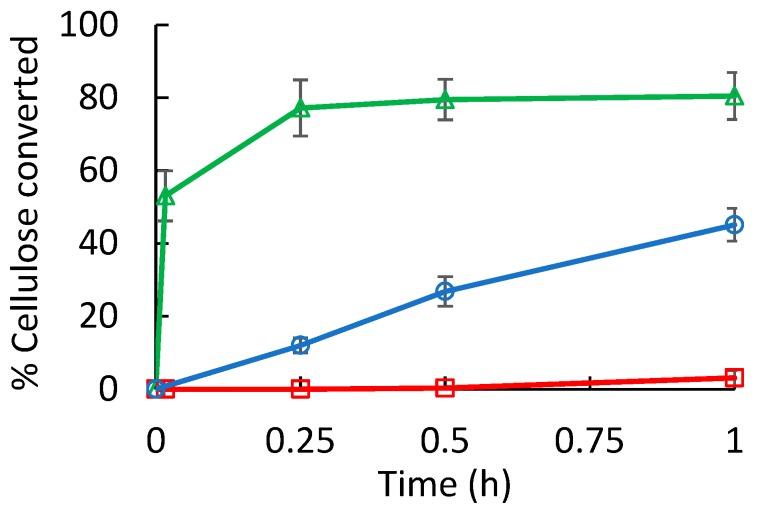
Hydrolysis of WSCA (∆) compared with that of microcrystalline cellulose (□) and cellulose hydrogel (○).

The foregoing results showed that WSCA dissolves in water, presenting in solution persistent chain segments (~10 nm) with an amphiphilic character. Enhanced enzymatic cellulose hydrolysis relies on disrupted crystalline packing of the cellulose chains presenting a high surface area exposed to water with some hydrophobic character for adsorption of the cellulose binding domains [37,38]. The hydrolysis rate of WSCA overwhelmingly increased as compared with microcrystalline cellulose and cellulose hydrogel particle dispersion (the fabrication of which has been reported previously [21]). In less than 5 min., more than 50 wt% of the cellulose was converted to reducing sugars. Hydrolysis rate was more than five times higher than the rate of samples prepared by the same procedure lacking the DCM [28,39], and hence lacking acetylation, as seen in Figure 7. This enhanced rate indicates that optimal conditions for enzymatic hydrolysis are achieved with water-soluble slightly acetylated cellulose.

## 4. Conclusions

Direct cellulose acetylation by its dissolution in EMIMAc/DCM mixture can be used to fabricate water-soluble cellulose acetate. The reaction product dissolves completely in water, with no significant signs of chain aggregation, and exhibits a large persistence length (>10nm), having an amphiphilic character. As a result, it is highly accessible to enzymatic hydrolysis, exhibiting an extremely rapid hydrolysis rate indicating its high availability for enzymatic absorption and reactivity.

## Figures and Tables

**Table 1:** Comparison of the experiment cellulose concentrations (wt%) vs. those calculated from fitting the SAXS measurements.

wt% Experimental	wt% Calculated
**2.5**	2.3
**2.0**	1.8
**1.5**	1
**1**	0.7
